# Active inference, morphogenesis, and computational psychiatry

**DOI:** 10.3389/fncom.2022.988977

**Published:** 2022-11-24

**Authors:** Léo Pio-Lopez, Franz Kuchling, Angela Tung, Giovanni Pezzulo, Michael Levin

**Affiliations:** ^1^Allen Discovery Center, Tufts University, Medford, MA, United States; ^2^Institute of Cognitive Sciences and Technologies, National Research Council, Rome, Italy; ^3^Wyss Institute for Biologically Inspired Engineering, Harvard University, Boston, MA, United States

**Keywords:** active inference, development, morphogenesis, defect, *Xenopus*, computational psychiatry

## Abstract

Active inference is a leading theory in neuroscience that provides a simple and neuro-biologically plausible account of how action and perception are coupled in producing (Bayes) optimal behavior; and has been recently used to explain a variety of psychopathological conditions. In parallel, morphogenesis has been described as the behavior of a (non-neural) cellular collective intelligence solving problems in anatomical morphospace. In this article, we establish a link between the domains of cell biology and neuroscience, by analyzing disorders of morphogenesis as disorders of (active) inference. The aim of this article is three-fold. We want to: (i) reveal a connection between disorders of morphogenesis and disorders of active inference as apparent in psychopathological conditions; (ii) show how disorders of morphogenesis can be simulated using active inference; (iii) suggest that active inference can shed light on developmental defects or aberrant morphogenetic processes, seen as disorders of information processing, and perhaps suggesting novel intervention and repair strategies. We present four simulations illustrating application of these ideas to cellular behavior during morphogenesis. Three of the simulations show that the same forms of aberrant active inference (e.g., deficits of sensory attenuation and low sensory precision) that have been used to explain psychopathological conditions (e.g., schizophrenia and autism) also produce familiar disorders of development and morphogenesis when implemented at the level of the collective behavior of a group of cells. The fourth simulation involves two cells with too high precision, in which we show that the reduction of concentration signaling and sensitivity to the signals of other cells treats the development defect. Finally, we present the results of an experimental test of one of the model's predictions in early *Xenopus laevis* embryos: thioridazine (a dopamine antagonist that may reduce sensory precision in biological systems) induced developmental (anatomical) defects as predicted. The use of conceptual and empirical tools from neuroscience to understand the morphogenetic behavior of pre-neural agents offers the possibility of new approaches in regenerative medicine and evolutionary developmental biology.

## 1. Introduction

Embryonic self-assembly requires large numbers of cells to cooperate and compete toward specific morphologies (Pezzulo and Levin, 2016). Moreover, the repair and remodeling of complex anatomical structures under novel circumstances (injury, changes in cell size or number, etc.) illustrates that this process is not hardwired. Numerous examples, such as tadpoles with scrambled craniofacial organs that move in un-natural paths to nevertheless construct normal frog faces (Vandenberg et al., 2012), and similar data even in early chordates (Voskoboynik et al., 2007), reveal control of growth and form to exhibit the ability to solve problems in morphospace—dealing with novel circumstances to arrive at the correct target morphology despite unexpected perturbations. It has been argued (Levin, 2019, 2022b) that morphogenesis is an example of a kind of basal intelligence, meeting William James' definition of “same ends through different means” (James, 1890), due to its ability to restore amputated structures (and then stop), produce the same size body using very large, or very few cells, or remodel after organs are placed in incorrect configurations (reviewed in Pezzulo and Levin, 2016; Levin, 2022a). Specifically, it has been proposed that the sorts of problem-solving capacities observed when nervous systems navigate the 3D world via behavior evolved by speed-optimizing much more ancient versions of the same system in pre-neural cells (Buznikov and Shmukler, 1981; Fields et al., 2020).

In an important sense, morphogenetic tissues and brains both represent a kind of collective intelligence, emergent from groups of cells (Fields et al., 2020; Fields and Levin, 2022). Much as neural cells connect in networks to process information and control muscles, collections of cells navigate anatomical morphospace by processing information toward the control of non-excitable cell types (resulting in cell migration, differentiation, etc.). Morphogenesis can thus be seen as the behavior of a collective in morphospace. Indeed, all of the machinery used in the central nervous system (CNS) to support adaptive functions (ion channels, electrical synapses, neurotransmitter signaling, etc.) is ancient, and is implicated in the control of growth and form (Levin and Martyniuk, 2018). Consistent with this are the many examples over the last decade of tools from neuroscience being used to probe and control animal shape *in vivo*: tools like optogenetics, ion channel pharmacology, etc. cannot distinguish neural from non-neural applications and work well in both arenas (Bates, 2015; Harris, 2021; Levin, 2021a). Given the high conservation between mechanisms regulating canonical behavior and those regulating morphogenesis (Pezzulo and Levin, 2015), it becomes interesting to ask: which conceptual models from neuroscience shed light on the collective dynamics of morphogenesis? This is especially important, both for regenerative medicine and for fundamental evolutionary developmental biology: progress on molecular genetics mechanisms must now be augmented by a better understanding of the algorithms and organ-level decision-making, to develop prediction and control strategies for large-scale structure and function. Here, we focus on one specific set of ideas from neuroscience—the active inference framework—Parr et al. (2022) exploring interesting parallels that allow it to generate insights for open problems in the science of dynamic morphogenesis.

What paradigm can be used to understand the dynamic information processing that underlies both, adaptive behavior and morphogenesis? Active inference is a general framework in theoretical neuroscience that proposes a unified account of perception, learning, and action (Friston et al., 2006; Friston, 2010). The framework is built around the idea that biological agents must maintain homeostasis and have to reach a limited range of states defined by their phenotype and avoid “surprising” states (Friston et al., 2010). However, as the states of the environment and the internal states of the organism itself are hidden (i.e., not directly observable), a biological agent must infer them from incoming sensory information. The framework claims that an agent is a probabilistic machine that embodies an internal generative model of its environment and continuously tries to minimize the surprise of its own sensations. In other words, an agent minimizes the difference between its expectations of the world (that can be priors on its own sensorium for perception, or goals for actions) and its sensory evidence. In turn, this requires that organisms continuously try to predict the internal (proprioceptive or interoceptive) and external (exteroceptive) consequences of their behavior and others—hence the idea of the brain as a “prediction machine.”

More formally, in active inference, action, perception, and learning occur through a process of variational free energy minimization (where variational free energy is an upper bound on surprise) (Friston K. et al., 2017). In this context, perception resolves exteroceptive prediction errors by selecting the predictions that best explain the current sensations; action serves to change the world in such a way that it generates the agent's preferred sensations (as encoded in its priors, or prior preferences); and learning is the update of the generative model.

**Figure 1:**
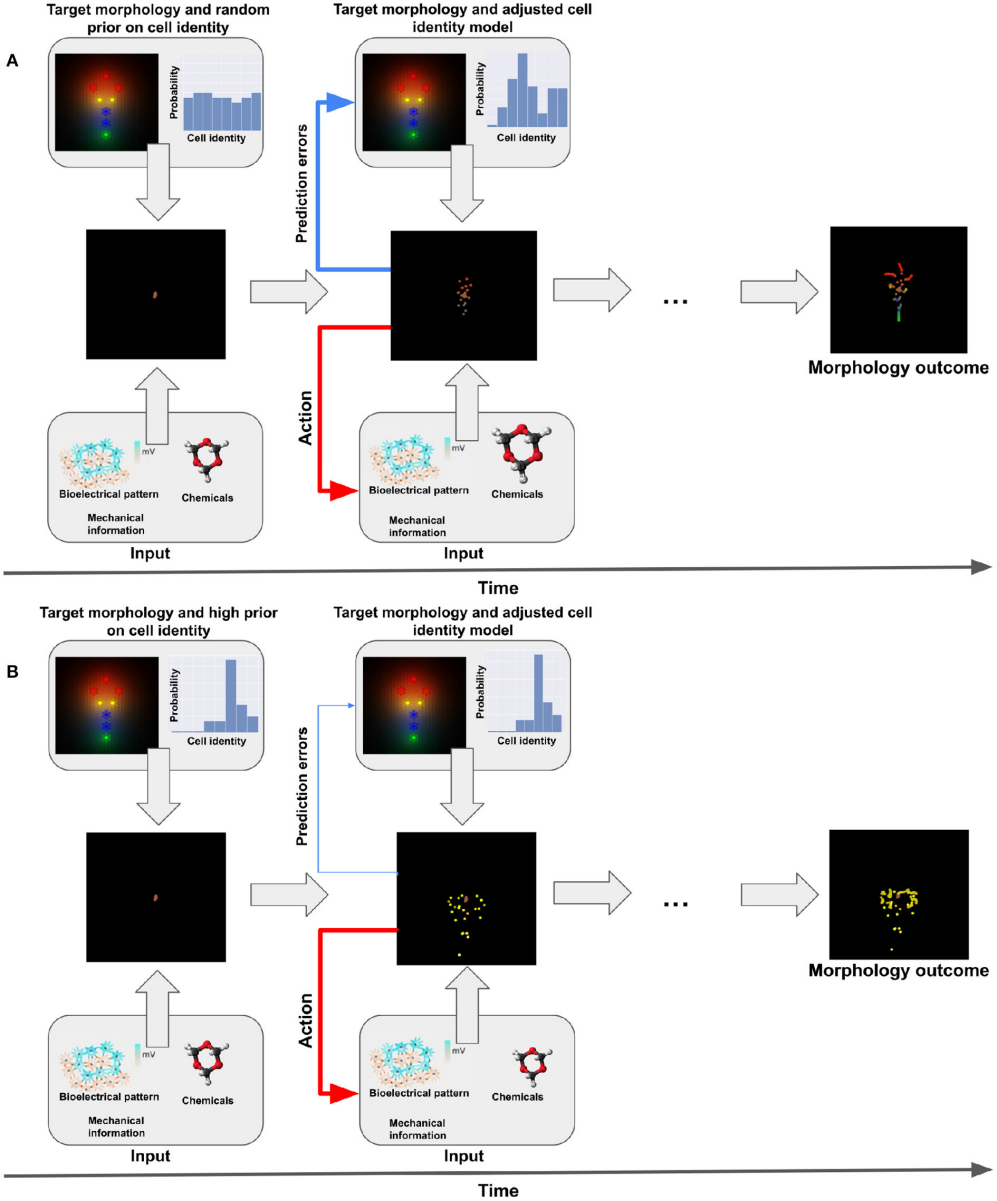
Representation of active inference. **(A)** Standard (non-pathological) behavior. We have one target morphology. During morphogenesis, cells start undifferentiated and at the same locations (middle-left panel). Given their generative model they believe they have to reach a specific planar target morphology (upper panel). They will receive different kind of sensory information during morphogenesis (bioelectrical pattern, biochemical and mechanical signals, see lower panel). This gathers sensory evidence that allows the cells to update their internal model of their target identity. Ultimately, they develop the appropriate target morphology (see middle-right panel) by action (migration, differentiation, release of signals, see second lower panel). **(B)** Dysfunctional behavior. In this case, the prior on the target morphology is much higher for one specific type of cells (intestinal in the example, see upper-left panel). The cells have a strong (rigid) belief that they have to be this type of cell and therefore won't take into account contrary sensory evidence (lower panel) and the prediction errors; in turn, this will lead to a dysfunctional update of the model and an inappropriate development (see middle panels).

The framework of active inference has been initially developed in computational neuroscience (Friston K. J. et al., 2017; Walsh et al., 2020) but was recently extended beyond it, to address problems of morphogenesis and developmental biology (Friston et al., 2015; Kuchling et al., 2020). In this perspective, cells are minimal active inference agents that minimize their surprise (or free-energy) in order to reach (collectively) a target morphology and maintain anatomical homeostasis (see Figure 1). This novel perspective on the coordination of migration and differentiation of cells suggests an interpretation of cellular behavior as being driven by an error minimization mechanism akin to usual conceptualizations of goal-directed organismal behaviors in neuroscience, cybernetics, and philosophy (Millikan, 1987; Friston et al., 2015; Fields and Levin, 2019; Kuchling et al., 2020). This top-down approach to developmental biology may complement bottom-up strategies that currently focus on molecular pathways and could help understand regeneration, morphogenesis, and their associated disorders.

In which sense can this framework help understand disorders of morphogenesis? Within the active inference framework, psychopathological conditions such as schizophrenia, hallucinations, and depression can be understood as disorders of inference (Montague et al., 2012; Friston et al., 2014). Along similar lines, active inference can help formalize developmental defects or aberrant morphogenetic processes as disorders of inference—when the inference is about (for example) a target morphology, as in Friston et al. (2015). Similarly, in developmental biology, focus is beginning to shift toward an understanding of measurement, memory, and prediction in cells and tissues (Barkai and Shilo, 2009; Abouchar et al., 2014; Cervera et al., 2019; Levin, 2021a).

The aim of this article is three-fold. Our first aim is to establish a formal link between disorders of inference apparent in psychopathological conditions and disorders of morphogenesis/development, via active inference. We exemplify our ideas by focusing mainly on schizophrenia and autism, because these conditions have been studied deeply under active inference and they depend on a key mechanism (aberrant precision control) that we believe could help explain developmental disorders, too (see below). However, we will not attempt to establish exact, one-to-one correspondences between these two (or other) psychopathological conditions and developmental disorders. Rather, our goals are to establish more general formal correspondences between psychopathological and morphogenetic/developmental problems—for example, using the concept of precision control that might be heuristic in both fields—and simultaneously to offer some specific examples, which could guide more specific future investigations. Our second aim is to show how disorders of morphogenesis can be simulated using active inference. Our third aim is to discuss how the formalization offered here could help design novel intervention strategies for repairing developmental defects or aberrant morphogenetic processes seen as disorders of information processing. We recognize that psychopathological conditions are very complex and multifarious (e.g., fundamentally shaped and constrained by complex cultural and sociological factors). The examples we are offering in this paper do not imply a reductionist view of these phenomena but are limited to modeling some specific computational aspects of them. Indeed, in this aspect our approach to cognition parallels the arguments made in our prior work about morphogenesis, where we suggest that a more effective understanding of collective cell behavior in embryogenesis and regeneration requires not only reductive molecular biology-based mechanistic information, but also a consideration of information processing and goal-directed action (Pezzulo and Levin, 2015; Levin, 2022a,c).

We present four simulations of disorders of morphogenesis as disorders of active inference. These simulations use active inference to model the behavior of groups of cells and show that endowing the cells with aberrant values of a key parameter (precision) leads to developmental defects in much the same way that manipulating the precision of more sophisticated active inference models could reproduce some aspects of psychopathological conditions, such as the positive symptoms of schizophrenia (Brown et al., 2013) or aberrant statistical learning in autism (Arthur et al., 2021). We also present a simulated biomedical intervention of the reduction of concentration signaling and sensitivity to the other cells signals of two cells having a too high precision. Finally, we present the experimental result of the test of one of the predictions of our model: thioridazine (a dopamine antagonist that may reduce sensory precision in biological systems) is shown to induced developmental (anatomical) defects in *Xenopus laevis* tadpoles, illustrating the ability of these conceptual models to drive novel empirical results in developmental biology.

## 2. Computational psychiatry, aberrant precision, and sensory attenuation

Active inference is a computational framework for action and perception and has been linked to various psychiatric disorders (Montague et al., 2012). From this point of view, psychopathological conditions can be understood as disorders of (active) inference. False inference could explain symptoms, like hallucinations or delusions in schizophrenia (Fletcher and Frith, 2009), to the loss of coherence in autism (Pellicano and Burr, 2012).

While there have been several alternative proposals about what exactly causes psychopathological conditions (Friston et al., 2014; Paulus et al., 2019; Van den Bergh et al., 2021), many focus on deficits of a specific parameter of the (active) inference: the *precision* parameter that weights prior beliefs and sensory evidence (and hence prediction errors, which measure the discrepancy between predicted and sensed observations). The precision parameter encodes the expected uncertainty or in other words the degree of confidence of an agent in the sensory information it is receiving in any given context (Angela and Dayan, 2005; Brown et al., 2013; Iglesias et al., 2013). A correct regulation of the precision parameter is key for the success of hierarchical (active) inference, because the balance between prior beliefs and sensory evidence during the process of evidence accumulation is determined by the precision of prediction errors at each level of the hierarchy. If the precision is set incorrectly (e.g., if an unreliable source of evidence is assigned excessively high precision, or a reliable source of evidence is assigned excessively low precision), the resulting inference can be misleading.

More formally, precision is defined in active inference as the inverse of the variance of a given variable; and it can be interpreted as a measure of signal-to-noise, or the confidence associated to an incoming information stream. For the brain, it can be represented by the modulation of the gain or excitability of neurons that compute prediction errors (Clark, 2013; Moran et al., 2013). This is particularly important, because many psychological disorders implicate modulatory neurotransmitter systems and a putative failure of post-synaptic gain control (Feldman and Friston, 2010).

One of the reasons why the precision parameter is so important in explaining psychopathological conditions is that it mediates the so-called sensory attenuation that necessarily accompanies action. In active inference, actions are realized by descending proprioceptive predictions, which in turn engage motor reflexes that enact the intended or predicted movement (Adams et al., 2013). In other words, in order to realize an action, an active inference agent has to imagine the action outcome and then enact a movement that fulfills the prevision. In parallel, it has to attenuate the sensory precision—in order to disregard the evidence that one is not moving. The attenuation of sensory precision is necessary for action, because otherwise, ascending (proprioceptive) prediction errors would lead to the revision of predictions about actions (i.e., correctly infer that one is not moving) instead of realizing the intended actions. Phenomenologically, the attenuation of sensory precision produces sensory attenuation—or the transient suspension of attention to the sensory consequences of an action—that is commonly observed during movement (Brown et al., 2013).

Sensory attenuation is an important theme in many psychiatric disorders, too. It has been proposed that prior expectations on sensory precision are compromised in schizophrenic patients—and hence the patients are unable to attenuate sensory precision. More specifically, the sensory precision of schizophrenic patients might be too high in relation to the precision of their (prior) beliefs about the causes of sensations. Therefore, schizophrenic patients cannot ignore sensory evidence. Deficits of sensory attenuation in schizophrenic patients may also produce disorders of movement, given that movement itself depends on correct precision control (see Brown et al., 2013 for a detailed discussion). The failure in sensory attenuation might explain two types of false inference in schizophrenia: false positives and false negatives (Limongi et al., 2018). Positive symptoms, like hallucinations or delusions (Powers et al., 2017), correspond to prior beliefs that are accorded too much precision. Negative symptoms, like the resistance to illusions and the failure of slow pursuit movements, correspond to a compromised capacity to elicit predictions informed by prior beliefs.

One symptom that shows this deficit is slow pursuit eye movements. Patients with schizophrenia present a disorder in the anticipation of movement. This failure can be measured experimentally using a mask during the motion of an object, with the subject having to anticipate the re-appearance of the object behind the mask. A computational model of this process that used active inference and manipulated precision could account for several features of smooth pursuit in schizophrenia: a reduction in anticipatory eye movements during visual occlusion, a paradoxical improvement in tracking unpredicted deviations from target trajectories, and a failure to recognize and exploit regularities in the periodic motion of visual targets (Adams et al., 2012).

Disorders of beliefs about agency are also common in schizophrenia (in the sense of agency as the property of feeling that one is the agent in control of one's actions). One example is the resistance of patients with schizophrenia to the force-matching illusion (Shergill et al., 2005, 2014). During this task, a patient's hand is touched by a device and therefore the patient can sense an external force. Patients have to press directly on themselves, or use a robot to reproduce the magnitude of the perceived pressure of the external force. The force-matching illusion consists in the fact that healthy people underestimate the magnitude of the force that they apply (i.e., self-pushing power), which results in pushing the device with a larger force than the external pressure. Patients with schizophrenia, instead, present a better accuracy on this task, which suggests that they might not attenuate their sensations. This result has been modeled using active inference and corresponds to an attenuation of the sensory precision that would create this illusion (Brown et al., 2013). Similarly, depersonalization disorder has been linked to a deficit of somatosensory attenuation and precision-weighting (Ciaunica et al., 2022).

Autism has also been linked to an aberrant account of precision (Lawson et al., 2014). Autism is a neurodevelopmental disorder of variable severity that is characterized by difficulties in social interaction and communication and by restricted or repetitive patterns of thought and behavior. Aberrant precision may explain different features of autistic perception, action, and social behavior. One hypothesis is that autism is characterized by excessively high sensory precision (compared to the precision of priors) leading to the difficulty in contextualizing sensory information (Frith and Frith, 1999; Baron-Cohen, 2000). Furthermore, in autism, the failure to attenuate sensory precision has been interpreted in a developmental context—and particularly in the context of the acquisition of generative models that distinguish between self and other. It is proposed that an infant with autism may have difficulties distinguishing between autonomic responses elicited by the mother and those caused by its own interoceptive predictions. This could also explain autonomic over-responsiveness to interoceptive cues or interoceptive hypersensitivity and a deficit in engaging with social (exteroceptive) cues (Paton et al., 2012).

In sum, here we have highlighted that psychopathological conditions, such as schizophrenia and autism, can be described in terms of aberrant values of key parameters of active inference, and most notably the precision parameter that weights sensory evidence and prior information [see also chapter 9 of Parr et al. (2022) for the discussion of other psychopathological conditions in terms of maladaptive active inference]. Below we highlight that these information- and system-level disorders have parallels outside of CNS-mediated behavior—in the domain of morphogenesis.

## 3. Disorders of morphogenesis as disorders of inference

A key fact of development and regeneration is that individual cells must cooperate in networks to achieve specific outcomes in morphospace, to very tight tolerances, despite noise, environmental influences, and imperfections in the components themselves. Doing this in real-time, to achieve the correct target morphology via novel paths, requires collective decision-making and active measurements of current shapes as well as the ability to recognize errors (perhaps through a memory of correct states). Morphogenesis has been simulated in the active inference framework (Friston et al., 2015; Kuchling et al., 2020). From this perspective, cells are information processing agents, where the driving force behind morphogenesis is the minimization of a cell's variational free energy. Each cell is equipped with a generative model that encodes its beliefs about what are the chemotactic signals it should receive or express, relative to its location in the target morphology. Cells reach their morphogenetic goal by performing actions (expression of receptors and other biochemical signals) according to their internal (i.e., generative) model of what type of receptors and other signals they should express.

Within this framework, Friston et al. simulated morphogenesis and dysmorphogenesis (e.g., induced birth defects) by manipulating the influence of extracellular signals, without changing the generative model (genetic and epigenetic processes) (Friston et al., 2015).

Using a similar strategy, Kuchling et al. simulated alterations of anterior–posterior axial polarity (i.e., the induction of two heads or two tails) as in planarian regeneration (Kuchling et al., 2020). Furthermore, they simulated the first steps of carcinogenesis by imposing false inference of a single cell on what it should sense and how it should act within the cellular ensemble. They also showed that mis-patterning of development and regeneration can be caused (or can be rescued) by simple modifications of the inference process without changing the implicit generative model of a cell as determined (for example) by its DNA.

However, the previous work on this topic did not investigate the impact of precision and sensory attenuation. In other words, the generative model had remained untouched in these prior simulations. Kuchling et al. manipulated external parameters to show disorders of morphogenesis (Kuchling et al., 2020), and Friston et al. (2015) manipulated the extracellular signals, both without changing the generative model or the precision of its parameters.

Here, we show that disorders of inference resulting from precision changes can produce disorders of morphogenesis in cell-like agents, analogous to the way aberrant precision control might produce psychopathological conditions in sophisticated (human-like) agents.

## 4. Active inference and free-energy minimization

### 4.1. Active inference formalism

In this section, we summarize the mathematical foundation that underlies the Bayesian interpretation of non-equilibrium steady-state dynamics [based on Kuchling et al. (2020)] and introduce the formulations necessary to set up and discuss our morphogenetic inference model based on the minimization of variational free energy.

To that end, we begin by stating the dynamics of our system in generalized coordinates of motion, denoted with a tilde, where $\tilde{x}$ is defined as:


(1) $$\begin{array}{l} \tilde{x}=(x,\dot{x},\overset{..}{x},...). \end{array}$$

This expression defines a state not just through its position, but also with its velocity, acceleration, etc. Generalized coordinates of motion allow the inclusion of temporal correlations in random fluctuations, assuming a sufficiently smooth dynamical system, through the Langevin equation:


(2) $$\begin{array}{l} \overset{\cdot }{\tilde{x}}=f\left(\tilde{x}\right)+\tilde{\omega }, \end{array}$$

where $f\left(\tilde{x}\right)$ is the generalized flow (or time evolution) of states under forces acting on the states and random fluctuations $\tilde{\omega }$ that are confined by the typical Wiener assumptions; in other words, the flow of states is made up of a process of independent, Gaussian increments that follow a continuous path.

In statistical physics, the ensuing dynamics are readily described in terms of density or ensemble dynamics, i.e., the evolution of the probability density $p\left(\tilde{x}\right)$, through the Fokker-Planck equation. We obtain the Fokker Planck equation from any Langevin equation using the conservation of probability mass:


(3) $$\begin{array}{l} \dot{p}(\tilde{x})=\nabla \cdot [\overset{\cdot }{\tilde{x}}p(\tilde{x})]=0, \end{array}$$

where $\overset{\cdot }{\tilde{x}}p\left(\tilde{x}\right)$ describes the probability current. This turns the Fokker-Planck equation into a continuity equation, which reads:


(4) $$\begin{array}{l} \dot{p}(\tilde{x})=\nabla \cdot \text{Γ}\nabla p-\nabla \cdot (f(x)p). \end{array}$$

This is a partial differential equation that describes the time evolution of the probability density $p\left(\tilde{x}\right)$ under dissipative (first term) and conservative (second term) forces. At non-equilibrium steady-state, the density dynamics is just the solution to the Fokker Planck equation:


(5) $$\begin{array}{l} L\left(\tilde{x}\right)=-lnp\left(\tilde{x}\right). \end{array}$$

In other words, we can express a physical potential or a Lyapunov function $L\left(\tilde{x}\right)$ simply as the negative log probability of finding the system in any (generalized) state $L\left(\tilde{x}\right)=-lnp\left(\tilde{x}\right)$. This is also known in information theory as the self-information of a state (also known as surprisal, or more simply surprise). In Bayesian statistics it is known as the negative log evidence.

This function can be bounded from above by the variational free energy function that is the foundation of the free energy principle underlying Bayesian inference.

Variational free energy is a function of internal states that allows one to associate the Lyapunov function with Bayesian model evidence and therefore characterize the system dynamics in terms of Bayesian inference and the implicit generative models. The key statistical mechanics tool employed to do this is to unpack the non-equilibrium steady-state flow of external, internal, and blanket states, called a Markov blanket partition.

A Markov partition, deriving from conditional independencies in Markov processes, which are implicit in the system's equations of motion or dynamics, separates all states *x*∈*X* into external *e*∈*E*, sensory *s*∈*S*, active *a*∈*A*, and internal states *i*∈*I* (with their generalized versions $\tilde{x},ẽ,\tilde{s},ã,$ and $\tilde{i}$, respectively), so that


(6) $$\begin{array}{l} \tilde{x}\in X=E\times S\times A\times I. \end{array}$$

The Markov blanket states separating external and internal states consist of *S*×*A*. Importantly, external and internal states depend only on the blanket states, with the further constraint that sensory states are not influenced by internal states and active states are not influenced by external states.

We note that the definition of a Markov blanket in a biological context is fairly intuitive, as robust literature demonstrates the ability of cells and many other aneural systems to measure aspects of their environment via specific sensors (Baluška and Levin, 2016). In fact, all biological systems can be analyzed in terms of sensory and internal states and the relationships between them (Rosen, 2012).

Following this Markov partition of states (and associated influences), we can decompose the flow $f\left(\tilde{x}\right)$ into $f_{e}\left(ẽ,\tilde{s},ã\right)$, $f_{s}\left(ẽ,\tilde{s},ã\right)$, $f_{a}\left(\tilde{s},ã,\tilde{i}\right)$, and $f_{i}\left(\tilde{s},ã,\tilde{i}\right)$.

We can derive a gradient descent for the Markov states using Equation (5) and its standard form expression for a flow *f*(*x*) subject to conservative and dissipative forces at non-equilibrium steady-state following (Yuan et al., 2014):


(7) $$\begin{array}{l} f(x)=v=(Q-\text{Γ})\nabla L(\tilde{x}), \end{array}$$

where Γ is the diffusion tensor defined as half the covariance of the dissipative random fluctuations, and a tensor *Q* describing friction satisfying $\nabla \cdot Q\nabla L\left(\tilde{x}\right)=0$. The responses of active and internal states, to sensory stimuli under Markov partition *m*, therefore, become


(8) $$\begin{array}{l} \left(a)f_{a}(\tilde{s},\tilde{a},\tilde{i})=(Q_{a}-{\text{Γ}}_{a})\nabla_{\tilde{a}}L(\tilde{s},\tilde{a},\tilde{i}\right)\\ \left(b)f_{i}(\tilde{s},\tilde{a},\tilde{i})=(Q_{i}-{\text{Γ}}_{i})\nabla_{\tilde{i}}L(\tilde{s},\tilde{a},\tilde{i}\right)\\ (c)L(\tilde{s},\tilde{a},\tilde{i})=-\ln p(\tilde{s},\tilde{a},\tilde{i}|m). \end{array}$$

Inserting the Lyapunov function from (c) into (a) and (b), gives us the resulting flow of active and sensory states as gradient descents on a log probability density:


(9) $$\begin{array}{l} \left(a^{\prime })f_{a}(\;\tilde{s},\tilde{a},\tilde{i})=({\text{Γ}}_{a}-Q_{a})\nabla_{\tilde{a}}\ln p(\tilde{s},\tilde{a},\tilde{i}|m\right)\\ (b^{\prime })\;f_{i}(\tilde{s},\tilde{a},\tilde{i})=({\text{Γ}}_{i}-Q_{i})\nabla_{\tilde{i}}\ln p(\tilde{s},\tilde{a},\tilde{i}|m). \end{array}$$

Crucially, the autonomous states (i.e., states that do not depend upon external states: active and internal) of an agent depend upon the same quantity, which we have reduced to the log probability of finding the agent in a particular state; where the agent's states consist of the internal states and their Markov blanket.

Solving Equation (9) for the time evolution or flow *f* of active and internal states thus is equivalent to evaluating the gradients of the log probabilities above, corresponding to the Lagrangian of an open system. By minimizing the internal and active states of the partition instead of minimizing the Lyapunov or Lagrangian function (such as for a thermodynamic potential) as would be done in a classical physics approach, we can now replace the Lagrangian with a variational free energy functional of a probabilistic model of how a system thinks it should behave, as follows.

Using the above Markov blanket partition, we can now interpret internal states as parameterizing some arbitrary probability density *q*(ẽ) over external states. This allows us to express the Lagrangian or Lyapunov function as a free energy functional of beliefs, and implicitly a function of the internal states. We can express this variational free energy through the introduction of the Kullback-Leibler Divergence:


(10) $$\begin{array}{l} D_{\text{KL}}(p||q)=\int_{-\infty }^{\infty }p(x)\ln\;\frac{p(x)}{q(x)}dx, \end{array}$$

which is the expectation of the logarithmic difference between the probabilities *p* and *q*, where the expectation is taken using the probabilities *p*.

Thus, instead of taking the log density $\ln\;p\left(\tilde{s},ã,\tilde{i}|m\right)$ above, we can now express a variational free energy *F* that corresponds to the logarithmic difference between the (variational) density or Bayesian beliefs about external states *q*(ẽ) and actual probability densities $p\left(ẽ,\tilde{s},ã,\tilde{i}|m\right)$ of all states under the Markov blanket *m*:


(11) $$\begin{array}{l} F(\tilde{s},\tilde{a},\tilde{i})=\int_{\tilde{e}}q(\tilde{e})\ln\frac{q(\tilde{e})}{p(\tilde{e},\tilde{s},\tilde{a},\tilde{i}|m)}d\tilde{e}\\ \;=-\ln p(\tilde{s},\tilde{a},\tilde{i}|m)+D_{\text{KL}}(q(\tilde{e})\|p(\tilde{e}|\tilde{s},\tilde{a},\tilde{i})). \end{array}$$

The first term is referred to as (Bayesian negative log) model evidence, or marginal likelihood, which denotes the likelihood that the sensory inputs were generated by a generative model implicit in the Markov blanket *m*. The second term is called relative entropy and works as to minimize the divergence between the variational and posterior density *q*(ẽ) and $p\left(ẽ|\tilde{s},ã,\tilde{i}\right)$, respectively. As a result, maximizing model evidence results in minimizing the free energy of the system, and because the divergence of the second term can never be less than zero, free energy is an upper bound on the negative log evidence. Using this expression, the flow of autonomous (i.e., active and internal) states becomes


(12) $$\begin{array}{l} \left(a^{\prime \prime })f_{a}(\tilde{s},\tilde{a},\tilde{i})=(Q_{a}-{\text{Γ}}_{a})\nabla_{\tilde{a}}F(\tilde{s},\tilde{a},\tilde{i}\right)\\ \;=({\text{Γ}}_{a}-Q_{a})\nabla_{\tilde{a}}\ln p(\tilde{s},\tilde{a},\tilde{i}|m)-({\text{Γ}}_{a}-Q_{a})\nabla_{\tilde{a}}D_{\text{KL}}\\ \left(b^{\prime \prime })f_{i}(\tilde{s},\tilde{a},\tilde{i})=(Q_{i}-{\text{Γ}}_{i})\nabla_{\tilde{i}}F(\tilde{s},\tilde{a},\tilde{i}\right)\\ \;=({\text{Γ}}_{i}-Q_{i})\nabla_{\tilde{i}}\ln p(\tilde{s},\tilde{a},\tilde{i}|m)-({\text{Γ}}_{i}-Q_{i})\nabla_{\tilde{i}}D_{\text{KL}}. \end{array}$$

Crucially, the gradient descent on variational free energy reduces the divergence in Equation (10) to its lower bound of zero (because the divergence cannot be less than zero). At that point, the gradients of the divergence in Equation (12) disappear and the dynamics reduce to the self-organization in Equation (9), which is what we aim to solve.

Now we can evaluate the variational free energy bound in Equation (11) in a straightforward way given a generative model; i.e., the joint probability over (generalized) external, internal, and blanket states. We can therefore associate the joint probability in Equation (11) with a likelihood; that is, the probability of a cell's states, given external states and a prior, in our case the prior probability of a cell's states (i.e., internal states and their Markov blanket). This means that *q*(ẽ) plays the role of a posterior density over hidden or external states under a particular Markov blanket or model (m). Importantly, this variational posterior is parameterized by internal states and we can talk about the internal states encoding beliefs about external states.

We now turn to the construction of this generative model, where we employ variational filtering as the method of quantification and minimization of a variational free energy, which places an upper bound on the dispersion of a particle's internal states and their Markov blanket (Friston, 2010; Buckley et al., 2017). This allows us to convert any process of self-organization into a gradient descent on a free energy landscape, where basins (minima) correspond to attractor states, or goal states—akin to the target morphology—as described next.

### 4.2. Active inference as a computational framework for morphogenesis

In this section, we introduce self organization to non-equilibrium steady-state under the lens of morphogenesis, using the variational principles described above. To that end, we simulated morphogenesis by specifying a generative model—and an implicit variational free energy function—and simulated self-organization by solving the equations of motion in Equation (12), as in Friston et al. (2015) and Kuchling et al. (2020).

Given a target morphology specified in terms of the location and differentiation of eight (undifferentiated) cells, we define a body morphology that the cells (collectively) have to reach, composed of a head, a body, and a tail (a single primary axis of positional identity, as exists for example in many metazoa). Migration and differentiation of each cell are key components of reaching a specific target morphology. In the active inference framework, it means that undifferentiated cells migrate and differentiate by minimizing free-energy. The dynamics of morphogenesis are mediated by chemotactic, biophysical, and electrochemical signals. Cell division is not taken into account in our model for the sake of simplicity. All cells are identical at the beginning of the simulation and they don't have any information on what kind of cell they are or where they are. Although they all have the same model, they are pluripotent and can differentiate into any kind of cell at the end.

In this multi-agent system, the active states of one cell (e.g., its secreted signaling molecules) are the external states to other cells (whose diffused concentrations it measures through it sensory states). Indeed, each cell has internal states and sensory states (of the Markov blanket) that correspond either to chemoreceptors of either extracellular and intracellular concentrations, while cell migration or the release of chemotactic signals are caused by the active states.

In terms of active inference, each cell is equipped with a generative model that encodes the beliefs it has about what chemotactic signals it should receive or express relative to its location in the target morphology.

We need to specify the generative model given by the probability density $p\left(\tilde{s},ã,\tilde{i}|m\right)$ of sensory states *s*, active states *a*, and internal states *i*, as well as the dynamics of the environment, determined through the flow *f*_ẽ_ and $f_{\tilde{s}}$ of external states *e* and sensory states *s*, respectively. This allows us to specify the requisite equations of motion for the system and its external states. Here, we will adopt a probabilistic nonlinear mapping with additive noise:


(13) $$\begin{array}{l} \begin{array}{cc} s=\; & g^{\left(1\right)}\left(e^{\left(1\right)}\right)+\omega^{\left(1\right)}\\ e^{\left(1\right)}=\; & g^{\left(2\right)}\left(e^{\left(2\right)}\right)+\omega^{\left(2\right)}, \end{array} \end{array}$$

where the superscripts denote the first and second levels of our hierarchical model *g*. Gaussian assumptions about the random fluctuations or noise ω mean that we can write the requisite likelihood and priors as:


(14) $$\begin{array}{l} \begin{array}{cc} p\left(\tilde{s},ã,\tilde{i}|{ẽ}^{1}\right)=\; & N\left(g^{\left(1\right)}\left(e^{\left(1\right)}\right),{\text{Π}}^{\left(1\right)}\right)\\ p\left({ẽ}^{1}|{ẽ}^{2}\right)=\; & N\left(g^{\left(2\right)}\left(e^{\left(2\right)}\right),{\text{Π}}^{\left(2\right)}\right). \end{array} \end{array}$$

where N is the normal distribution, and Π^(*t*)^ denotes the precision (or inverse variance) of the random fluctuations.

We then construct the approximate posterior density *q*(ẽ) introduced in Equation (10) using the associated Lagrangian or Lyapunov function


(15) $$\begin{array}{l} L(\tilde{x})=-\ln p(\tilde{s},\tilde{a},\tilde{i},\tilde{e}|m)\\ \;=-\ln p(\tilde{s},\tilde{a},\tilde{i}|{\tilde{e}}^{1})-\ln p({\tilde{e}}^{1}|{\tilde{e}}^{2}). \end{array}$$

Under a Laplace assumption, the variational density becomes a normal distribution:


(16) $$\begin{array}{l} q\left(ẽ\right)=N\left(\tilde{i},-\nabla_{\tilde{i}\tilde{i}}L\left(\tilde{s},ã,\tilde{i},\tilde{i}\right)\right), \end{array}$$

where $\nabla_{\tilde{i}\tilde{i}}L\left(\tilde{s},ã,\tilde{i},\tilde{i})\right)$ denotes the curvature of the Lagrangian with respect to internal states. With this generative model and assumed form for the variational density, we can now evaluate the variational free energy for any given sensory state and perform a gradient descent according to Equation (12).

An agent minimizing variational free energy is essentially updating beliefs of its environment and itself, under the generative model, so that the evolution of the system will inevitably lead to a non-equilibrium steady state of minimal free energy. Again, this appears rather intuitive in a developmental or regenerative biological context: cells function to remodel tissues and organs, in order to minimize the global difference between the current configuration and a species-specific anatomical goal state (Pezzulo and Levin, 2015; Pezzulo and Cisek, 2016). Such cellular behavior is based on perceived signals from their environment, and act with respect to expectations that are genetically encoded, and shaped by cellular learning (Baluška and Levin, 2016).

As free energy corresponds to (an upper bound on) Bayesian model evidence $-\ln\;p\left(\tilde{s},ã,\tilde{i}|m\right)$ as introduced in Equation (11)—the ensuing self-organization is also self-evidencing. Self-evidencing in neuroscience refers to the idea that the brain is separated from its environment by a “statistical boundary,” which can be formally described by the statistical construct of a Markov blanket (Kirchhoff et al., 2018). We note that such a description of a system's dynamics in Bayesian terms [such as Bayesian beliefs *q*(ẽ)] and self-evidencing is a purely technical formulation based on the underlying mathematical definitions within the framework, which can be ascribed to simple systems like macromolecules and cells, as opposed to just higher cognitive organisms.

The simulations in this work encompass a small set of cells that are each equipped with the same generative model—such that they collectively self-organize to minimize variational free energy in an interdependent way, which has all the characteristics of morphogenesis. This example is appropriate to models such as the highly-regenerative planaria (Durant et al., 2016; Levin et al., 2019), which we will use multiple times in illustrating the principles in this work.

**Figure 2:**
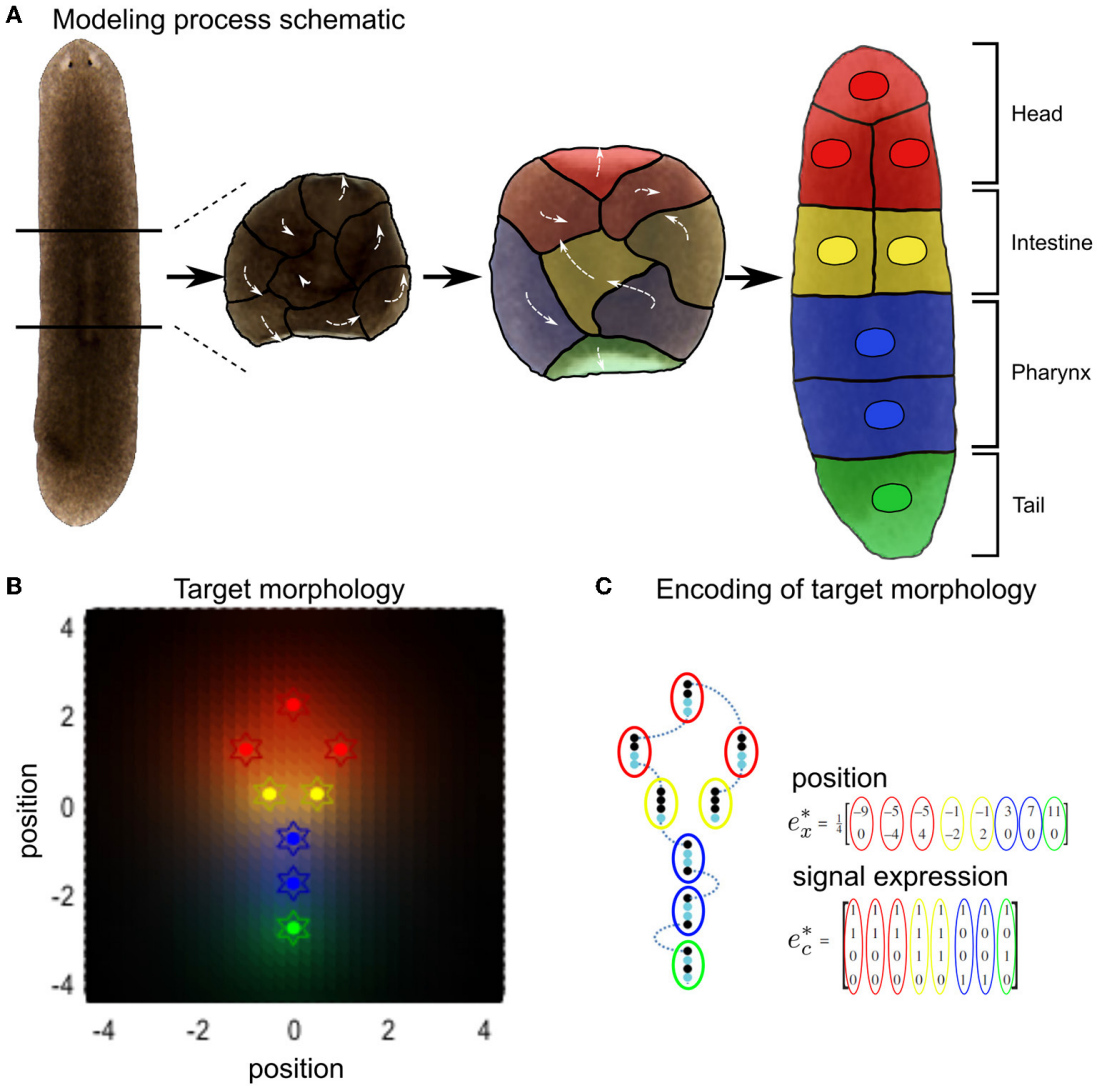
Schematic of variational Bayesian simulation of simple body shape morphogenesis illustrated by the example of regenerative patterning observed in planarian flatworms. **(A)** When dissecting out the center piece of a planarian flatworm, the constituent cells will remodel into a whole new worm with normal body axis. For the purpose of illustrating our simulation and for simplicity, cells that form different tissue types were grouped together as one cell. **(B)** Expected Signal concentrations (background color hues) at each target final position (colored stars) in the target morphology encode the cellular model of inference, with the same color encoding cell type from **(A)**. **(C)** The target morphology for the arrangement of cells is encoded by expectations of external signals $e_{c}^{*}$ for any given position $e_{x}^{*}$ in the defined target morphology that constitutes the final configuration of cells. Each row in $e_{c}^{*}$ corresponds to a different signaling type, while every column represents the signal expression states for a different cell. Adapted from Kuchling et al. (2020).

All the cells in our simulations are initialized with random signaling profiles near the center of their environment. The morphogenetic task of self-organization to a target configuration thus corresponds, in this framework, to each cell inferring its own location within the ensemble by forming and testing its beliefs (or predictions) *q*(*e*) about the hidden causes of the signaling concentrations it senses (i.e., the signaling molecule secretion profiles and hence cell identities of the other cells (Figure 2).

The active states a cell has control are the type of signals it can secrete, and its movement in any direction in 2D. Furthermore, each cell possesses a place-encoded model of a target configuration common to all cells based on signaling concentrations that would be sensed under that configuration. Therefore, for each of the four possible cell types corresponding to specific positions in the cell cluster, cells expect to sense specific concentrations of signaling molecules.

Sensory states *s* are therefore chemotactic concentrations of intracellular, exogenous and extracellular signals:


(17) $$\begin{array}{ll} s & =\left[\begin{array}{c} s_{c}\\ s_{x}\\ s_{\lambda } \end{array}\right]=\left[\begin{array}{c} e_{c}\\ e_{x}\\ \lambda \left(e_{x},e_{c}\right) \end{array}\right]+\omega , \end{array}$$

where *e* are the external states of concentrations *c* and positions *x* of other cells. The signal concentration *s*_λ_ at each position of the *i*-th cell is given through the secretion and diffusion of signaling molecules of each other cell *j* and of itself, determined by the coefficient:


(18) $$\begin{array}{l} \lambda_{i}\left(e_{x},e_{c}\right)=\tau \cdot \underset{j}{\sum }e_{cj}\cdot exp\left(-kd_{ij}\right). \end{array}$$

Here, *e*_*cj*_ is the combination of the four signals expressed at each position *j*, depicted in Figure 2B as color coded around the target positions e^*^, that are defined in Figure 2C, and


(19) $$\begin{array}{l} d_{ij}=|e_{xi}-e_{xj}|, \end{array}$$

is the distance between the *i*-th cell and the remaining cells, to which the secreted signal diffuses with diffusion coefficient *k*.

Analogous to stem cell-like behavior, we specify the same generative model *g* for each cell:


(20) $$\begin{array}{ll} g\left(e\right) & =\left[\begin{array}{c} e_{c}^{*}\\ e_{x}^{*}\\ \lambda^{*} \end{array}\right]\sigma \left(e\right), \end{array}$$

where $\lambda^{*}=\lambda \left(e_{c}^{*},e_{x}^{*}\right)$ is the signal concentration at the target locations, and


(21) $$\begin{array}{l} \sigma (e_{j})=\frac{\exp e_{j}}{\sum_{j}\exp e_{j}} \end{array}$$

is the softmax function (or normalized exponential). This is a commonly utilized function in neural networks to enforce a sum to one constraint, which allows an interpretation as a categorical distribution over mutually exclusive outcomes.

Using these expressions—and the equations of motion from the previous section—we can express the flow of internal and active (*i.a*. autonomous) states from Equation (12) as


(22) $$\begin{array}{l} (a^{\prime \prime })\;f_{a}(\tilde{s},\tilde{a},\tilde{i})=(Q_{a}-{\text{Γ}}_{a})\nabla_{\tilde{a}}F(\tilde{s},\tilde{a},\tilde{i})=D\tilde{a}-\nabla_{\tilde{a}}\tilde{s}\cdot {\text{Π}}^{\left(1\right)}\tilde{\epsilon }\\ (b^{\prime \prime })\;f_{i}(\tilde{s},\tilde{a},\tilde{i})=(Q_{i}-{\text{Γ}}_{i})\nabla_{\tilde{i}}F(\tilde{s},\tilde{a},\tilde{i})=D\tilde{i}-\nabla_{\tilde{a}}\tilde{\epsilon }\cdot {\text{Π}}^{\left(1\right)}\tilde{\epsilon }\\ \;-{\text{Π}}^{\left(2\right)}\tilde{i}, \end{array}$$

while suppressing higher order terms. Here, ϵ = *s*−*g*(*i*) is the prediction error associated with sensory states—the state of chemotactic signal receptors—and can hence be expressed as:


(23) $$\begin{array}{ll} \epsilon & =\left[\begin{array}{c} \epsilon_{c}\\ \epsilon_{x}\\ \epsilon_{\lambda } \end{array}\right]=\left[\begin{array}{c} s_{c}-e_{c}^{*}\sigma \left(i\right)\\ s_{x}-e_{x}^{*}\sigma \left(i\right)\\ s_{\lambda }-\lambda^{*}\sigma \left(i\right) \end{array}\right]. \end{array}$$

D is the matrix derivative operator on generalized states and the signal precision Π^(1)^ is set to 1. We assumed Gaussian priors (with a mean of 0) over the hidden states with a small precision Π^(2)^ (i.e., high variance) with a log precision of minus two.

With this generative model in place, the internal states organize themselves to minimize (precision-weighted) prediction error based upon predictions of sensed signaling states from neighboring cells. In neuroscience, this scheme is also referred to as predictive coding and can be viewed as a generalized form of Bayesian (variational) filtering as described earlier. Predictive coding describes the dynamics of the system in terms of prediction errors ϵ through accumulation of model evidence $lnp\left(\tilde{s},ã,\tilde{i}|m\right)$, which maximizes likelihood $p\left(\tilde{s},ã,\tilde{i}|{ẽ}^{1}\right)$ (Friston et al., 2009). This is exactly the type of process underlying the formulation of variational free energy above.

Matlab software running these simulations, under different conditions can be downloaded as part of the academic SPM software from https://www.fil.ion.ucl.ac.uk/spm/software/ (accessed via a graphical user interface invoked with the command > > DEM). The code of the simulations can be found at https://github.com/LPioL/active_inference_morphopsy/.

## 5. Developmental defects as the result of a deficit of sensory attenuation or aberrant precision

Here, we use active inference to simulate dysmorphogenesis and disorders of sensory attenuation, similarly to what we discussed above, in relation to schizophrenic and autistic patients. We first present the standard (non-pathological) simulation of morphogenesis by setting appropriate values of sensory and prior precisions. Then, we show and analyze three simulations of aberrant morphogenesis. The first simulation illustrates the effects of high sensory precision in driving aberrant morphogenesis, analogous to what is observed in negative symptoms of schizophrenia and autism. The second simulation illustrates the effects of excessive prior precision in driving aberrant morphogenesis, analogous to so-called positive symptoms of schizophrenia (e.g., hallucinations and delusions, Powers et al., 2017). The third simulation illustrates the effects of low sensory precision in driving dysmorphogenesis, which might be analogous to forms of hyporeactivity.

### 5.1. Standard (non-pathological) morphogenesis

**Figure 3:**
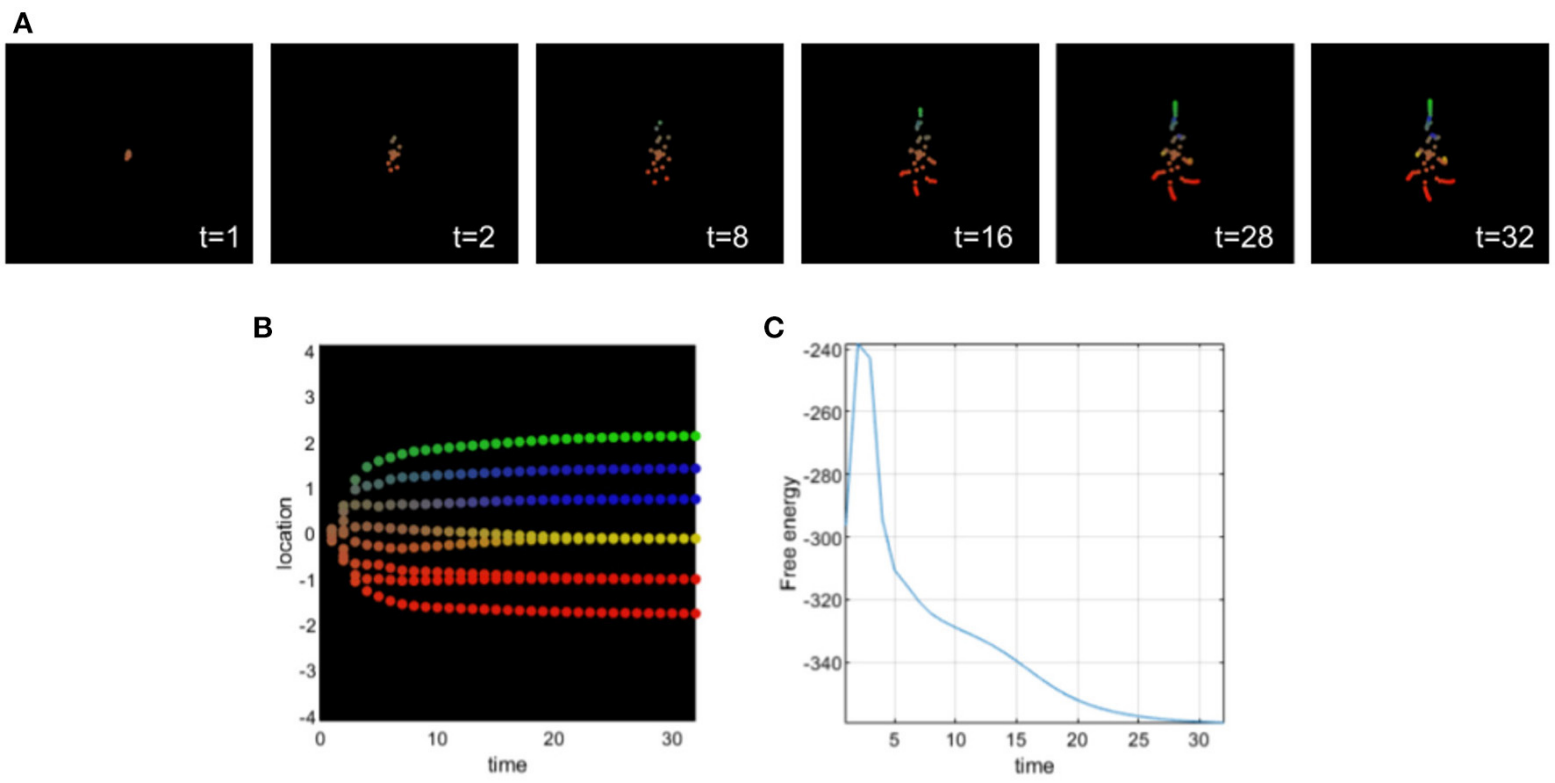
**(A)** Time-lapse movie montage of simulation of normal morphogenesis. The eight initially unspecified cell types perform chemotaxis and update their posterior beliefs in order to infer the correct target morphology. **(B)** Differentiation profile of the eight cells during morphogenesis. **(C)** Free-energy minimization during morphogenesis.

Setting appropriate values for the precision terms leads to normal morphogenesis and the normal differentiation and migration of cells, which reach the appropriate location in the morphospace. The cells correctly infer their locations by forming predictions about the hidden causes of the signaling concentrations (without any perturbation of their precision parameters) and they reach the target morphology (see Figure 3).

In this simulation, ascending prediction errors are associated with an appropriate sensory precision, and sensory signals are processed in a context-sensitive way leading to an appropriate collective inference.

### 5.2. Excessively high sensory precision in morphogenesis

**Figure 4:**
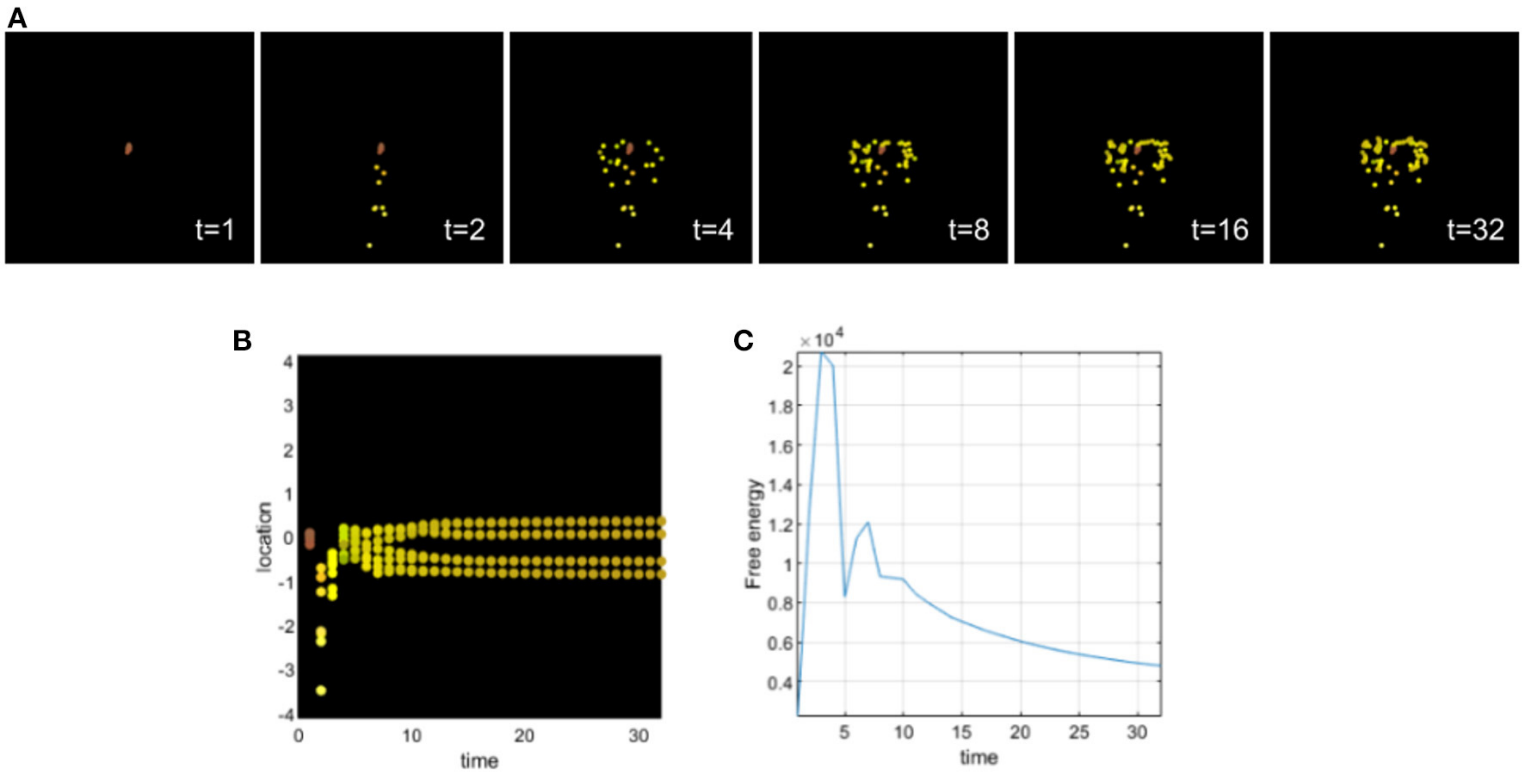
**(A)** Time-lapse movie montage of simulation of morphogenesis with a deficit of sensory attenuation high precision on the sensory signals received by the cells. The eight initially unspecified cell types perform chemotaxis and update their posterior beliefs in order to infer the correct target morphology. **(B)** Differentiation profile of the eight cells during morphogenesis. **(C)** Free-energy minimization during morphogenesis.

This simulation illustrates a deficit of sensory attenuation (analogous to what is observed in autism and schizophrenia). For this, we assigned an excessively high precision to the biochemical signals sensed by all cells (see Figure 4), i.e., a value that greatly exceeds the optimal value in this case. An excessively high sensory precision will increase the influence of ascending prediction errors of sensory channels in which the cells will place more confidence. We can observe that during differentiation, all cells become intestinal cells and they migrate without following the body plan of the planaria; in other words, the cells tend to stay around a circle. By assigning a high sensory precision, the cells develop a deficit of sensory attenuation and do not behave properly. They secrete chemicals as if they were already intestinal cells and the collective organization and communication fail to give them the appropriate information necessary to reach the appropriate morphology. Sensory prediction errors stay high during development, and top-down prediction errors are not taken into account by the cells leading to this development defect. Cells only integrate local cues, and high sensory precision leads to their inability to contextualize sensory information. This behavior renders sensory prediction errors too precise and context insensitive as top-down information won't be taken into account.

This example illustrates a possible development of a homogeneous tumor from a deficit of sensory attenuation. The majority of the cells stay close to each other and do not migrate appropriately. We can also observe some cells that migrate very fast at the beginning of the morphogenesis. We can draw a parallel in development with neurocristopathies (Bolande, 1974). Neurocristopathies are a developmental defect that are characterized by the abnormal specification, migration, differentiation, or death of neural crest cells during embryonic development. High sensory precision leads to increased estimates of and larger updates about environmental volatility as is sometimes observed in relation to autism (Lawson et al., 2017). Excessively precise systems (including cells) try to extract too much information from noisy signals (or too close signals), instead of treating noise as irreducible. This implies that such a system will update its generative model frequently, without improving its estimate over time. The system remains with some unaccounted uncertainty and this may be stressful in biological systems looking for homeostasis.

**Figure 5:**
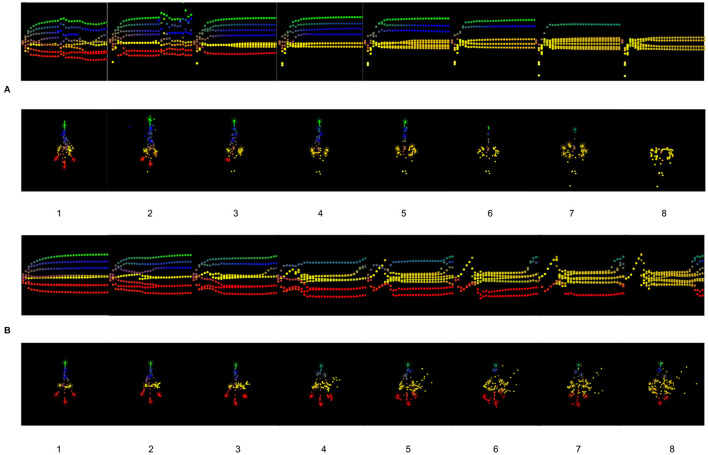
**(A)** Results of the simulations for one to eight cells having a too high precision. **(B)** Results of the simulations for one to eight cells having a too high prior on their identity, the cells think they are intestinal cells.

In Figure 5A, from two cells having too high precision, we have a developmental defect where one head (red) cell can't reach the appropriate location, but instead stays close to intestinal cells and doesn't completely differentiate. With the increasing number of cells having too high precision, the development of the tumor composed only of intestinal cells is also increasing. The cells fail to infer their location and identity as a collective, they are no longer able to communicate and infer with the other cells.

### 5.3. Excessively high prior on the identity of cells in morphogenesis

**Figure 6:**
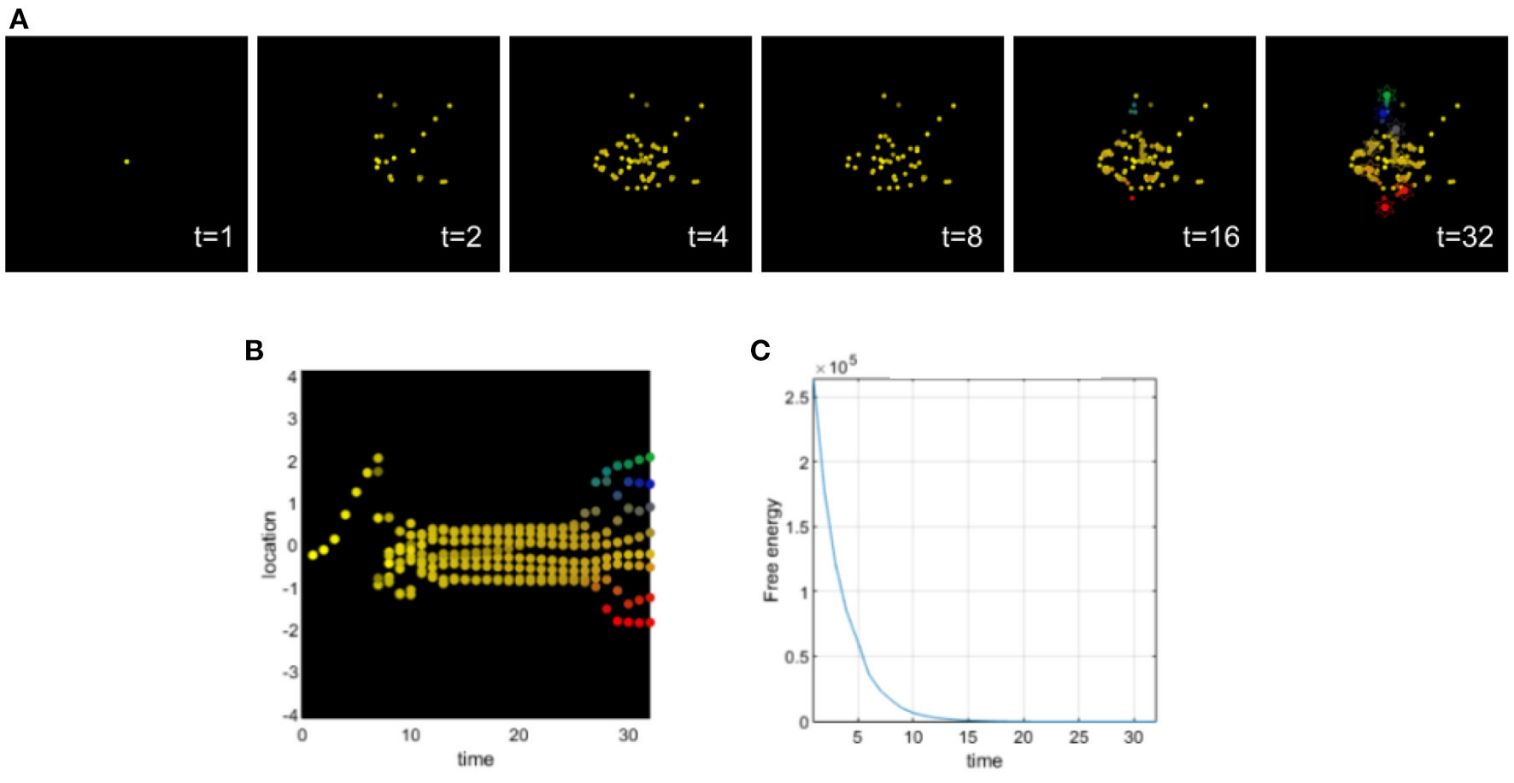
**(A)** Time-lapse movie montage of simulation of morphogenesis with a deficit of sensory attenuation via high prior on identity of the cells. The eight initially unspecified cell types perform chemotaxis and update their posterior beliefs in order to infer the correct target morphology. **(B)** Differentiation profile of the eight cells during morphogenesis. **(C)** Free-energy minimization during morphogenesis.

This second simulation illustrates the effects of assigning an excessively high prior on the identity of the cells analogous to what is hypothesized for the positive symptoms of schizophrenia (see Figure 6). Too strong of a prior makes the cells not accept contradictory information effectively, sensory attenuation is too high reducing their ability to handle novel circumstances (such as regenerative repair). Here, all cells believe before differentiation and migration that they are intestinal cells. We can observe that at the beginning of morphogenesis (see Figure 6), cells are confused and migrate in all directions and mostly at the center as if they were intestinal cells. The differentiation in intestinal (yellow) cells last 28 steps of the simulation and then the cells undergo a partial differentiation to their appropriate identity trying to recover the target morphology. We can observe that the cells of the head and tail do not migrate perfectly to the target morphology. Their location and their differentiation are incomplete. Biologically, this resembles the remodeling of scrambled “Picasso head” in tadpoles, with eyes, jaws, and other organs in the wrong locations, into normal frog faces (Levin, 2021a).

This example also illustrates the pluripotential capacities of the cells and the ability of self-assembly to recover from drastic changes in precision and sensory attenuation.

Similar to the previous simulation, we can observe an abnormal migration of cells as in neurocristopathies that result from a derangement of neural crest migration, colonization, or differentiation (Bolande, 1974, 1997).

As we can observe in Figure 5B, from four cells with a too high expectation on their identities, we can observe incomplete differentiation of several cells. After a period of time, cells recover the target morphology with respect to the migration to the appropriate locations, but the differentiation is not complete. There is an important developmental noise, which leads to a disorder of inference for the other cells in terms of differentiation.

### 5.4. Excessively low sensory precision in morphogenesis

**Figure 7:**
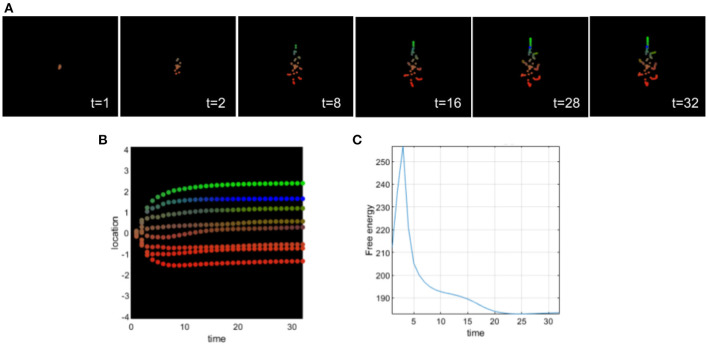
**(A)** Time-lapse movie montage of simulation of morphogenesis with a deficit of sensory attenuation via low sensory precision of the cells. The eight initially unspecified cell types perform chemotaxis and update their posterior beliefs in order to infer the correct target morphology. **(B)** Differentiation profile of the eight cells during morphogenesis. **(C)** Free-energy minimization during morphogenesis.

This simulation illustrates the effects of setting a very low sensory precision for all cells in the collective (see Figure 7). A low sensory precision relative to the precision of prediction errors higher in the hierarchy, will bias perception toward prior beliefs, which in this case are randomly initialized.

We can observe that intestinal cells do not successfully differentiate; this is indicated by the fact that they remain brown throughout the entire simulation. Only one pharynx cell (blue) differentiates properly. The other pharynx cell is between an undifferentiated state (brown) and a tail state (green). Overall the cells didn't migrate to the exact location of the target morphology, but this brown-green incompletely differentiated pharynx cell deviated the most from its target.

Low sensory precision is associated with hyporeactivity (Idei et al., 2021). In this case, the cells would not react enough to the chemicals they were sensing, leading to incomplete differentiation and migration of several cells of the collective; this in turn leads to failure of anatomical maturation.

### 5.5. Simulated biomedical intervention to rescue a collective with two cells having too high sensory precision

**Figure 8:**
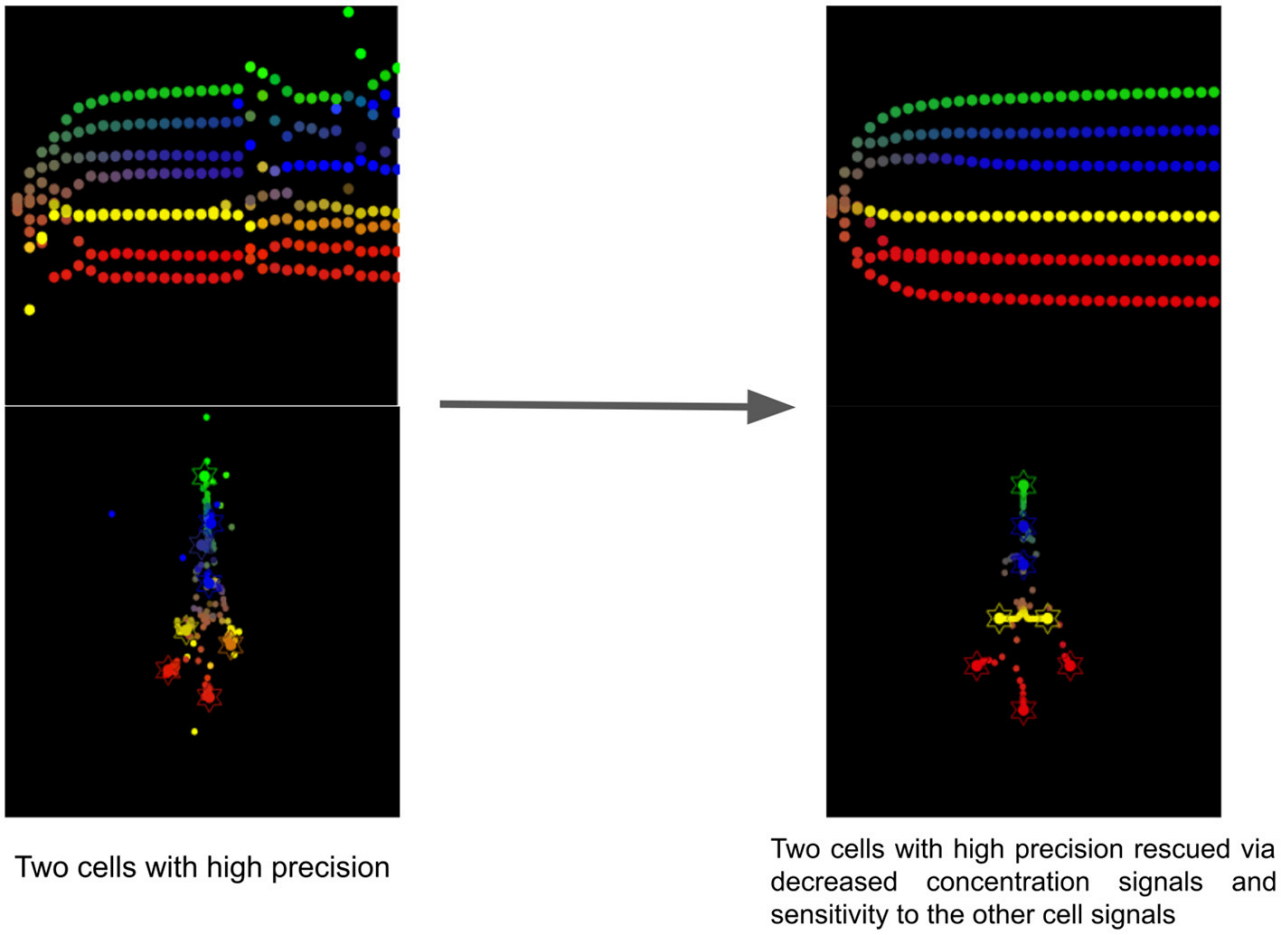
Timelapse of the rescue of two cells with too high precision via reduction of concentration signals and sensitivity to cell signals of the other cells.

This simulation shows the effect of the reduction of concentration signaling and sensitivity to the other cells signals of the two cells having a too high precision (see Figure 8). Without this intervention, the collective is unable to reach the target morphology and one head (red) cell stays close to intestinal cells and never fully differentiates. After the simulated biomedical intervention, as we can observe in Figure 8, the developmental defect is cured and all cells reach their location with the appropriate differentiation. The developmental noise we can observe in the pathological case is also removed.

This resembles the effects of antipsychotics for the treatment of schizophrenia. Most of them are antagonists of D2 receptors and therefore reduce the sensitivity of neurons to dopamine (Seeman and Kapur, 2000). In our simulation, we reduced the sensitivity to the other cell signals for the two cells having a too high precision, suggesting that dopaminergic drugs acting as antagonists could be tested in morphogenesis. Indeed, in active inference, the precision of sensory information is assumed to be mediated by neuromodulators, such as dopamine (Friston et al., 2012) or acetylcholine (Vossel et al., 2014).

## 6. Experimental test of the dopamine antagonist thoridazine on the development of *Xenopus laevis* embryos

**Figure 9:**
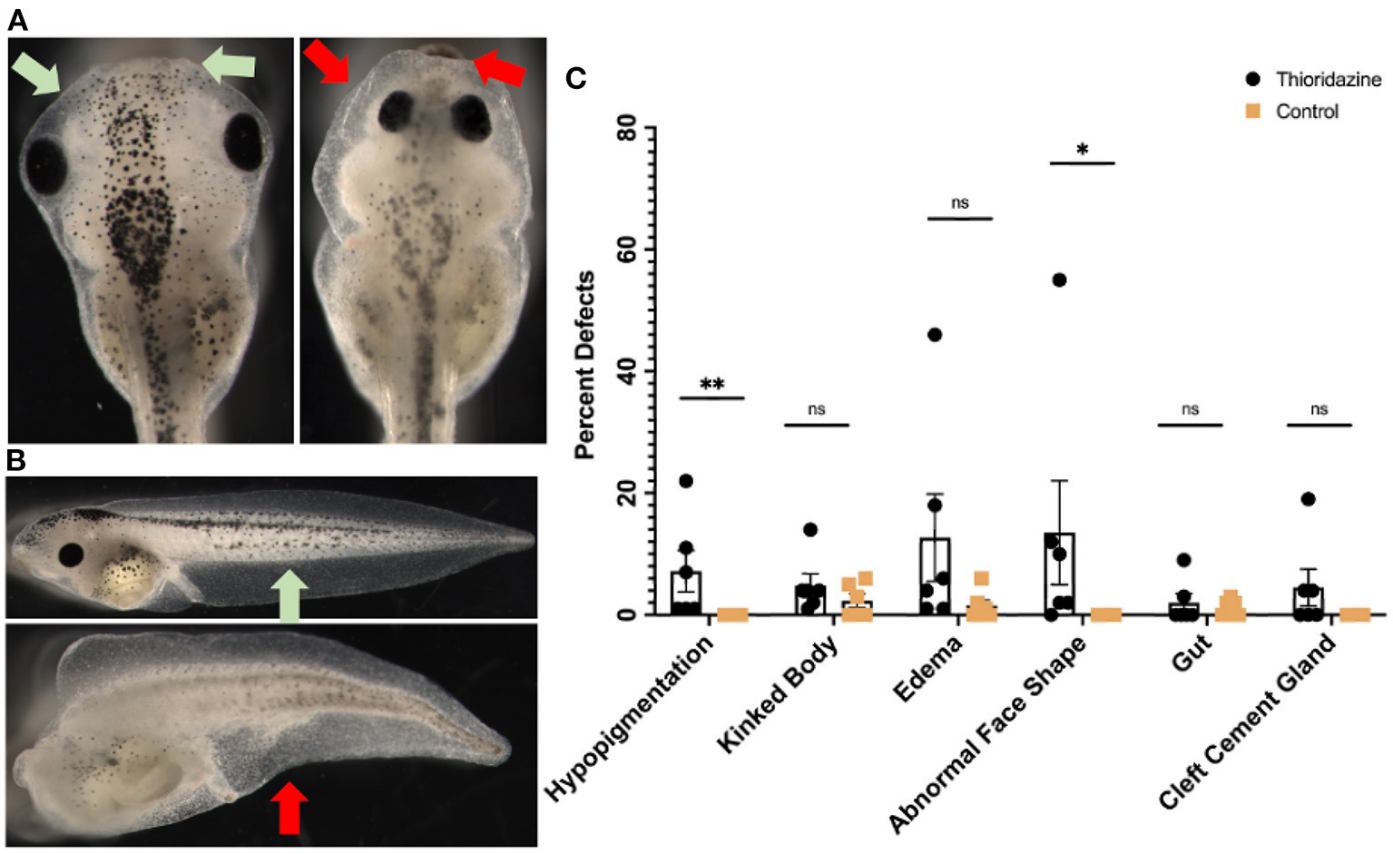
Experimental results showing the effects of thioridazine on the development of *Xenopus laevis* embryos. Compared to normal embryos in **(A)**, treated embryos exhibit significant levels of abnormalities that include hypopigmentation, kinked bodies, edemas, abnormal face shapes, malformed guts, and cleft cement glands **(B)**. The percent of embryos that had each defect is shown in **(C)**. Treated embryos had higher incidents of hypopigmentation (7.17%), edemas (12.67%), abnormal face shapes (13.5%), and cleft cement glands (4.5%) than their control counterparts, summarized in **(C)**. Each point represents one separate trial of 100 embryos each and is graphed as a mean with SEM and **p < 0.01 and *p < 0.05.

Our framework emphasizes strong parallels between the dynamics of cognition and those of collective cell behavior during embryogenesis and regeneration. Due to evolutionary conservation (Fields et al., 2020), these parallels are not only functional but are mechanistic: the same machinery used to drive information processing in the brain should be implicated in the control of anatomical development. While this first generation of such models do not have the detail to be able to predict precisely that anatomical outcome of specific perturbations, they do make a surprising prediction not implied by other existing models of developmental events: that perturbation of dopamine signaling will cause errors in morphogenesis, including of non-neural tissues. Thus, we next tested a prediction of our models—that finely-tuned levels of sensory precision in cells are necessary for correct embryogenesis—using a loss-of-function pharmacological assay in early frog embryos. Thioridazine is a dopamine receptor antagonist and has been used to treat schizophrenia (Fenton et al., 2007). In the active inference framework, as a dopamine receptor antagonist, this drug may reduce sensory precision in biological systems (Friston et al., 2012) and according to our simulations this drug should induce different developmental defects. Thus, we applied this drug to *Xenopus laevis* embryos during early stages (see section 6) and scored their morphogenesis by the swimming larva stage. Compared to normal embryos (Figures 9A-left, Figures 9B-top), treated embryos exhibited abnormalities that included hypopigmentation, kinked primary axes, edemas, abnormal face shapes, malformed guts, and cleft cement glands (Figures 9A-right, Figures 9B-bottom). The percentage of embryos with each defect is shown in Figure 9C; raw counts are shown in Table 1.

**Table 1:** Raw counts of thioridazine induced defects in six separate trials of 100 embryos.

	**Thioridazine**						**Control**					
Hypopigmentation	7	1	1	22	1	11	0	0	0	0	0	0
Kinked body	4	4	4	1	2	14	0	0	6	5	3	0
Edema	4	1	1	46	18	6	0	0	6	2	1	0
Abnormal face shape	12	2	2	55	0	10	0	0	0	0	0	0
Gut	9	0	0	0	0	3	2	0	3	0	2	0
Cleft cement gland	0	4	4	0	0	19	0	0	0	0	0	0

These results are consistent with our models, which suggest that modulation of mechanisms, such as neurotransmitter signaling (known to be involved in information processing in the field of behavioral and brain sciences), can regulate morphogenesis of non-neural structures. More broadly, these results contribute to the study of morphogenesis as behavior of a collective cellular agent in anatomical morphospace (Fields and Levin, 2022).

### 6.1. Experimental methods

#### 6.1.1. Animal husbandry

*Xenopus laevis* tadpoles were raised in 0.1X Marc's Modified Ringers solution (MMR), pH 7.8, and were staged according to Nieuwkoop et al. (2020). All embryos (pooled from separate female frogs and then randomly divided) were raised at 14°C. Feeding stage animals were fed three times a week with Sierra Micron powdered diet, and media changes were performed on alternate days. Tadpoles were raised at a density of 100 tadpoles per 40 ml of media in 100 × 20 mm petri dishes. All experiments were approved by the Tufts University Animal Research Committee (M2020-35) in accordance with the guide for care and use of laboratory animals.

#### 6.1.2. Pharmacological exposures

Thioridazine (thioridazine-HCl, Sigma-Aldrich) was dissolved in deionized water at 1mM, and were then frozen in single use aliquots to prevent continuous re-thawing. Drug exposure occurred at 90 μM in 0.1X MMR conducted on NF stage 12.5–26 embryos that had been reared at 14°C prior to exposure. During the treatment, experimental and controls embryos were kept at 18°C. After the exposure they were returned to 14°C until they reached scoring stage 45. Scoring was performed by inspection of morphology using transmitted light microscopy.

## 7. Discussion

In this article, we simulated disorders of morphogenesis as disorders of (collective) inference. The decision of one cell is a function of the states of the other cells; or in other words, the collective sends the appropriate signals to each individual cell. In a functional sense, the collective “remembers” the body shape; if one cell diverges, the collective continues emitting signals that guide that cell until it again reaches the correct position. We implemented deficits of sensory attenuation and aberrant precision that are usually invoked as explanations of autism and schizophrenia and also considered a low precision simulation that has been linked to hyporeactivity. All of these simulations lead to developmental defects via aberrant sensory precision relative to the prior precision. We presented a proof of concept appealing to disorders of inference and showed that precision control is useful in understanding not only psychopathological conditions, but also developmental defects and morphogenesis (see Table 2).

**Table 2:** Table of the associations between active inference, psychopathology, and dysmorphogenesis.

**Active inference**	**Psychopathology**	**Dysmorphogenesis**
Excessively high sensory precision	Autism Deficit of sensory attenuation like in schizophrenia Larger update about environmental volatility	Homogeneous tumor Neurocristopathies Total disorganization
Excessively high prior (e.g., on belief states or the identity of cells)	Deficit of sensory attenuation like in schizophrenia	Organs in the wrong place (Robin and Nadeau, 2001) Not enough of one organ Incomplete differentiation
Excessively low sensory precision	Hyporeactivity	No differentiation of some cells (not enough growth) Deficits of migration Wrong organ

We have furthermore shown how manipulations of the information processing modes, in terms of precision and prior strength, interplay with biochemical signals in developmental biology, such as signaling ligand concentration and receptor sensitivities. Crucially, our (simulated) results showed that the disrupting effect of individual cells' signaling aberrations on the overall morphology can be rescued by decreasing the diffusion of signaling ligands and receptor sensitivities, an effect that would have been hard to predict without our proposed view of certain morphological defects as disorders of inference and precision control (Pezzulo and Levin, 2015). This simulated result remains to be experimentally tested *in vivo* and is left as future work. This new framework that focuses on the precision of spatiotemporal signal progression during development comes at an opportune moment, when tools such as optogenetics (Bugaj et al., 2017) and microfluidics (Sonnen and Merten, 2019) have provided us for the first time with enough spatiotemporal precision to study and manipulate these events in real time on a single cell level.

If developmental defects are the results of disorders of active inference similar to what we can find in psychopathologies like schizophrenia or autism, it may be possible that the psychoparmacology used for hyper- or hyporeactivity, hallucinations, or other symptoms related to these diseases could be used to address developmental defects. Various case studies highlight this possibility. Indeed, by applying different reagents targeting the glutamatergic, adrenergic, and dopaminergic pathways to *Xenopus laevis* embryos from gastrulation to organogenesis stages, Sullivan and Levin observed numerous developmental defects, including craniofacial defects, hyperpigmentation, muscle mispatterning, and miscoiling of the gut (Sullivan and Levin, 2016). Serotonin pathways are also implicated in left-right asymmetry and the pre-neural morphogenesis (Fukumoto et al., 2005; Levin et al., 2006). Serotonin signaling has also been linked to autism and may be involved in this pathology during early brain development (Yang et al., 2014). In addition, usually, dopamine, serotonin, and adrenaline receptors, are molecular targets for antipsychotics and schizophrenia (Stepnicki et al., 2018); and we have now provided evidence that modulators of decision-making in traditional behavioral systems have predicted effects on morphogenetic behavior of cell collectives. Therefore, by targeting similar pathways during development, we may be able to reverse developmental defects.

These ideas are part of a bigger effort to solve the inverse problem in regenerative medicine (Lobo et al., 2014): strategies such as CRISPR and genome editing cannot reach their full potential because it is usually too difficult to know what to change at the hardware level (DNA and pathways) to achieve desired changes at the level of anatomy. Fortunately, neuroscience has blazed the trail in a pluralistic understanding of complex systems: work in that field (and types of intervention targets) ranges across studies of ion channel protein structure, synaptic plasticity, network roles in image processing and memory, behavioral responses, and psychological drives and analysis. Neuroscience would greatly benefit from understanding how non-neural cells work, because neurons share many molecular and functional properties with non-neural cells. Thus, while current biomedical approaches have focused entirely on manipulating the lowest level (gene regulatory networks and protein structure/pathways), we propose to begin to deploy interventions across scales in regenerative medicine, as commonly occurs in the behavioral sciences. We have previously proposed that high-level manipulation of tissues' perceptions and memories (via training for example) may be a better way to manipulate large-scale properties of morphogenesis in health and disease than bottom-up micromanagement of the protein-level hardware (Pezzulo and Levin, 2015; Mathews and Levin, 2018).

The active inference framework is a very powerful aspect of system-level functionality in cognition (Friston K. et al., 2017; Pezzulo et al., 2018; Parr et al., 2022), and a key target of strategies to exploit invariants between neuroscience and other fields (Friston, 2013; Fields and Levin, 2020; Fields et al., 2021). Thus, once we reach a better understanding of the role of precision in development and morphogenesis, it may be possible to apply “reverse inference” as we can see for computational psychiatry (Schwartenbeck and Friston, 2016). Reverse inference in development/morphogenesis reflects the probability of a developmental process being present given the knowledge of activation in a particular tissue region. The aim is to infer from a specific anatomy which precision parameters caused it (i.e., which precision parameters were incorrectly set). Similarly to Schwartenbeck and Friston (2016) where the researchers infer the model parameters of a single subject or a group of subjects from measured behavioral or neuronal responses, it could be possible to develop a “computerized clinical diagnosis” for developmental defects leading to specific biomedical interventions. These are just the first steps on a roadmap to regenerative medicine that borrows from neuroscience the highly fruitful approaches focused on modulation of systems at different levels, from molecular pathways to behavioral decision-making. A future computational somatic psychiatry will take advantage of the tools of behavioral and cognitive sciences to manipulate not only the hardware of cellular pathways, but also the decision-making of cellular collectives. By taking seriously the native competencies of tissues (Levin, 2021b, 2022c), it may eventually be possible to effectively modulate outcomes in birth defects, traumatic injury, and cancer, not by micromanagement but by quantitative behavior shaping in morphospace.

## Data availability statement

The original contributions presented in the study are included in the article/supplementary materials, further inquiries can be directed to the corresponding author.

## Author contributions

LP-L and FK wrote code, performed computational experiments, and analyzed data. AT performed *Xenopus* experiments and analyzed data. GP and ML developed the project, guided the experimental design and conceptual foundations, and provided oversight and funding. All authors contributed intellectually to the work, co-wrote the manuscript, and approve the submitted version.

## Funding

This research received funding from the European Union's Horizon 2020 Framework Programme for Research and Innovation under the Specific Grant Agreement No. 945539 (Human Brain Project SGA3) to GP and the European Research Council under the Grant Agreement No. 820213 (ThinkAhead) to GP. ML gratefully acknowledges support by the Templeton World Charity Foundation (grant TWCF0606) and the John Templeton Foundation (grant 62212).

## Conflict of interest

The authors declare that the research was conducted in the absence of any commercial or financial relationships that could be construed as a potential conflict of interest.

## Publisher's note

All claims expressed in this article are solely those of the authors and do not necessarily represent those of their affiliated organizations, or those of the publisher, the editors and the reviewers. Any product that may be evaluated in this article, or claim that may be made by its manufacturer, is not guaranteed or endorsed by the publisher.
